# Inferior Oblique Muscle Overaction: Clinical Features and Surgical Management

**DOI:** 10.1155/2019/9713189

**Published:** 2019-07-17

**Authors:** Ercan Ozsoy, Abuzer Gunduz, Emrah Ozturk

**Affiliations:** ^1^University of Health Sciences, Haseki Training and Research Hospital, Department of Ophthalmology, Istanbul, Turkey; ^2^Inonu University School of Medicine, Department of Ophthalmology, Malatya, Turkey

## Abstract

**Purpose:**

To further define the clinical features of patients with inferior oblique muscle overaction (IOOA) and evaluate the surgical results in a subgroup of these patients.

**Methods:**

The medical records of 173 patients who underwent inferior oblique muscle (IO) weakening surgery due to primary or secondary IOOA were retrospectively reviewed. The patients were assigned a surgical group based on severity of IOOA and presence of dissociated vertical deviation (DVD) or hypertropia. Patients with +1 or +2 IOOA underwent recession, patients with +3 or +4 IOOA underwent myectomy, and patients with any grade of IOOA and DVD or hypertropia underwent anterior transposition (AT) surgery.

**Results:**

A total of 286 eyes of 173 patients who underwent surgery due to IOOA were included in the study. IOOA was accompanied by esotropia, exotropia, abnormal head posture (AHP), pattern strabismus, convergence insufficiency, DVD, facial asymmetry, and nystagmus. The most common comorbid disorder was esotropia. The recession was used in 173 eyes, myectomy in 64, and AT in 49. Surgical success was obtained in 96.0% of eyes that underwent recession, in 98.4% of eyes that underwent myectomy, and in 93.9% of eyes that underwent AT. In the follow-up, IOOA occurred in the fellow eye in 36.1% of patients who underwent unilateral surgery.

**Conclusions:**

This study is a comprehensive report on the concomitants of the IOOA. Also, it showed that all of the three surgical procedures including recession, myectomy, and AT are effective in the surgical management of IOOA when performed in select patient groups.

## 1. Introduction

Inferior oblique muscle overaction (IOOA) manifests by overelevation of the eye in adduction and is frequently associated with horizontal deviations. It is reported in 70% of patients with esotropia and 30% of patients with exotropia. There are two types of IOOA: primary and secondary. Primary type is frequently bilateral and its etiology is unclear, but secondary type is unilateral and is caused by ipsilateral superior oblique (SO) palsy or contralateral superior rectus palsy [1–4].

Clinical features of primary type include elevation of the eye on adduction, slight vertical deviation in the primary position, minimal head tilt, and a negative Bielschowsky test, whereas the features of secondary type include increased vertical deviation in the primary position, elevation of the paralyzed eye on adduction, marked head tilt, and a positive Bielschowsky test [5, 6].

Surgical weakening of the inferior oblique muscle (IO) is performed either unilaterally or bilaterally because of functional and/or aesthetic reasons in treatment of the primary IOOA or secondary IOOA due to SO palsy. Various surgical procedures have been described to weaken the IO, including tenotomy, myotomy, myectomy, recession, extirpation-denervation, hang-back recession, nasal transposition, muscle fixation, anterior transposition (AT), and graded AT [4, 7–9]. There is currently no consensus on the best surgical procedure for weakening of the IO.

The purpose of this study is to describe the clinical features of patients with IOOA and to evaluate the results of three different surgical procedures which were selected based upon the severity of IOOA and the amount of vertical deviations.

## 2. Patients and Methods

We retrospectively reviewed the medical records of 173 patients who underwent the IO weakening surgery due to primary or secondary IOOA between 2012 and 2017. An informed consent form had been obtained from the patients or their parents before the surgery. We conformed to the tenets of Helsinki Declaration (1964) during the study. Patients with a follow-up period of 6 months or longer were included in the study. Patients with a history of other ocular diseases or previous extraocular muscle surgery were excluded from the study.

A detailed history was obtained from all patients and followed by a complete eye examination including cycloplegic refraction, best spectacle-corrected visual acuity (BSCVA), fundus examination, and measurement of the deviation in diagnostic gaze positions at near and at distance by prism and alternate cover test. The severity of IOOA was graded from 0 to +4 as follows: (0) no IOOA, (+1) mild upwards deviation of the pupil from the horizontal line in adduction, (+2) upper margin of the pupil becomes aligned to the margin of the upper lid in adduction, (+3) superior half of the pupil covered by the upper lid in adduction, and (+4) the entire pupil covered by the upper lid in adduction.

Patients were assigned a surgical group based on severity of IOOA and presence of DVD or hypertropia. Patients with +1 or +2 IOOA underwent recession, patients with +3 or +4 IOOA underwent myectomy, and patients with any grade of IOOA and DVD or hypertropia underwent AT surgery. In patients associated with horizontal deviations, the IO weakening surgery was performed along with the horizontal strabismus surgery. The amount of horizontal surgery was independent of the IO weakening surgery.

## 3. Surgical Techniques

All cases were operated under general anesthesia by the same surgeon. In three procedures, initially a 8 mm postlimbal incision was made in the inferotemporal quadrant and the IO was found and isolated with a muscle hook. At the conclusion of the case, the conjunctival wound was closed with interrupted 8-0 polyglactin sutures.

### 3.1. Recession Technique

A 6-0 double-needle polyglactin suture was placed at its distal end, and the IO was detached from the sclera. Following, the IO was sutured to the sclera 3 mm posterior and 2 mm lateral to the temporal edge of the inferior rectus muscle (IR) insertion which equals to 10 mm of recession.

### 3.2. Myectomy Technique

The IO was cut at the insertion site and a 8–10 mm segment of the intracapsular portion of the muscle was removed. The muscle was released into the muscle sheath.

### 3.3. AT Technique

A 6-0 double-needle polyglactin suture was placed in the muscle near its insertion, following the muscle was cut from its insertion. The IO was sutured to the sclera 1 mm lateral to the temporal border of the IR insertion site at five different points which ranged from 2 mm anterior of the insertion site to 2 mm posterior with a 1 mm of space. In all eyes, the IO was reattached to the sclera without spreading at the new insertion site and the new IO insertion line was oriented perpendicular to the IR axis. The amount of anteriorization depended on the severity of IOOA and the amounts of DVD or hypertropia in the primary position.

In the recession and myectomy groups, a successful outcome was defined as a grade 0 of IOOA at 6 months postoperatively, and in the AT group, a grade 0 of IOOA with a DVD or hypertropia <5 PD was defined as a successful outcome at 6 months postoperatively.

## 4. Statistical Analysis

The SPSS software version 22.0 (SPSS Inc., Chicago, IL, USA) was used for statistical analysis. The *x*^2^ and Mann–Whitney *U* tests were used for data analysis. The *P* value of <0.05 was considered to be significant.

## 5. Results

A total of 286 eyes of 173 patients with IOOA included in this study. Demographic and ophthalmologic examination findings of the patients with IOOA are summarized in Table 1. The mean follow-up period was 11.0 (6–72) months.

There was no statistically significant difference for mean age, gender distribution, and spherical equivalent between the three surgical groups (*P* : 0.068, *P* : 0.40, and *P* : 0.529, respectively). There was a statistically significant difference for the postoperative mean follow-up time between the recession and AT groups (*P*=0.041).

In 146 compliant patients, the mean preoperative BSCVA was 0.78 ± 0.29 in the decimal system. The mean preoperative BSCVAs of recession, myectomy, and AT groups are 0.78 ± 0.30, 0.69 ± 0.30, and 0.86 ± 0.23, respectively. There was a statistically significant difference in the mean preoperative BSCVA between recession and myectomy groups (*P*=0.034) and also between myectomy and AT groups (*P*=0.004).

V pattern was diagnosed in 35 patients (20.2%), dissociated vertical deviation (DVD) in 11 (6.3%), nystagmus in 7 (4.0%), and facial asymmetry in 7 (4.0%). Asymmetric IOOA was observed in 32 of 90 patients (35.5%) with bilateral IOOA.

The mean preoperative hypertropia in 38 patients who underwent AT surgery was 15.97 ± 4.91 prism diopters (PD), and the mean preoperative DVD in 11 patients who underwent AT was 14.09 ± 7.98.

**Figure 1:**
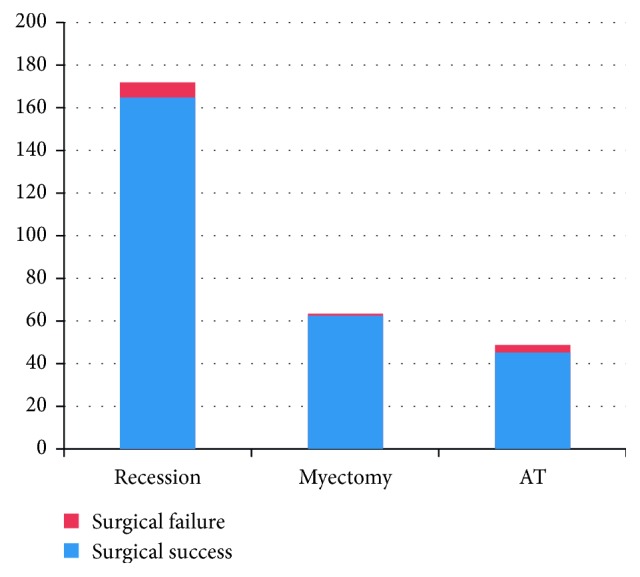
Surgical success and failure of the groups. AT: anterior transposition.

Recession was performed in 173 (60.5%) eyes, myectomy in 64 (22.4%), and AT in 49 (17.1%). After surgery, IOOA developed in the unoperated fellow eye in 30 (36.1%) of 83 patients who underwent surgery due to unilateral IOOA. Twenty-three (76.6%) of these 30 patients underwent IO weakening surgery in the fellow eye. In the postoperative period, the development of IOOA in the fellow eye was significantly correlated with primary type, small mean age, and long follow-up time (*P*=0.008, *P*=0.001, and *P*=0.001). Concurrent horizontal rectus muscle surgery to correct an associated horizontal deviation was performed in 129 patients (74.5%). The rates of surgical success in each surgical group are presented in Table 2.  Figure 1 shows success and failure in each surgical group.

In the sixth month of the postoperative period, IOOA persisted in 11 patients (3.8%), AHP in 6 patients (3.4%), pattern strabismus in 3 patients (1.7%), and convergence insufficiency in 9 patients (5.2%). Hypertropia and DVD were less than 5 PD in all patients in the AT group. There were no serious complications other than a conjunctival cyst in 2 patients (1.1%) who underwent recession and antielevation syndrome in 2 patients (1.1%) who underwent AT, and in one of them, the IO had been sutured to the sclera 1 mm anterior to the IR insertion and in the other 2 mm.

## 6. Discussion

This study retrospectively evaluated 286 eyes of 173 patients who underwent IO weakening surgery due to primary or secondary IOOA. We detected esotropia in 53.8% of patients with IOOA, followed by orthotropia in 25.4% and exotropia in 20.8%. There was a predominance for esotropia in the primary position, which was consistent with previously reported rates of IOOA in 70% of patients with esotropia and in 72% of patients with congenital esotropia [2, 10].

We found AHP in 21.9% of patients with primary type and in 64.5% of patients with secondary type. A study reported AHP in 78.8% of patients with unilateral SO palsy, and another study reported in 55.5% of patients [11, 12]. Our result on the frequency of AHP in secondary type is between these reported rates.

Pattern deviations were diagnosed in 20.2% of patients with IOOA in the present study. All of the patients with pattern strabismus had a V pattern. Although the reported whole prevalence of pattern deviations in strabismic patients is between 12.5% and 87.7%, about 20% of strabismic patients might be expected to have a pattern deviation [13–15]. Our result of patterns' frequency supports these previous findings.

Recession of the IO is one of the two most commonly performed procedures for weakening of the IO [16]. Patients with low-grade IOOA can be treated by recession of the IO [6]. Parks compared recession, disinsertion, myectomy between origin and IR, and myectomy at insertion in patients with bilateral IOOA without hyperdeviation and reported recession to be the most effective method for treatment of IOOA. In his study, the IO was reattached to the sclera in a point 2 mm temporal and 3 mm posterior to the insertion of the IR [17]. In another study, the IO was recessed to a point 2 mm lateral and 4 mm posterior to the insertion of IR in 40 eyes to treat IOOA and the recession was reported to be effective in the surgical management of IOOA [18].

In the recession group, we obtained a surgical success in 166 of 173 eyes (96.0%). Our criterion for a surgical success was an IO function of grade 0 at 6 months postoperatively. In 7 eyes (4%), residual IOOA was detected. Parks reported postoperative residual overaction in 19% of patients who underwent bilateral IO recession surgery [19]. This finding is higher than our outcome on the frequency of residual overaction. Although Parks recessed the IO to the point as we did, this difference may arise from the grades of IOOA in Parks's study. Parks performed the recessions in patients with any grade of IOOA and without hyperdeviation and we performed the recessions only in patients with +1 or +2 IOOA and without hyperdeviation.

Myectomy is the another commonly used procedure in the surgical management of IOOA. Myectomy procedure is mainly done for +3 IOOA [16, 20]. In a study, myectomy was performed in 42 eyes and recession in 40 eyes for treatment of IOOA and both procedures were reported to be effective for weakening of the IO with similar success rates. In the aforementioned study, the patients with IOOA were randomly assigned to either myectomy or recession group, and in the myectomy group, a 5 mm portion of the IO was removed [18]. Another study compared disinsertion, myectomy, and AT for treatment of primary or secondary IOOA and reported a success rate of 91.7% in the disinsertion group, 97.8% in the myectomy group, and 89.5% in the AT group with minimum side effects. In the mentioned study, the surgical procedure was determined according to the surgeon's preference, and in the myectomy group, a 5 mm segment or more was removed from the IO [1].

In the myectomy group, we obtained a surgical success in 63 of 64 eyes (98.4%) when a satisfactory result is considered as no hyperfunction in the IO at 6 months postoperatively. In this group, although some eyes showed IO underaction in the early postoperative time, this dysfunction disappeared in the first 6 months after the surgery in all eyes. In one eye (1.5%), overaction of the IO persisted. In two previous retrospective studies, postoperative overacting IO rates have been reported to be 1.7% and 5% after myectomy surgery [21, 22]. Our finding on the frequency of the IOOA persistence is similar to these previously reported outcomes.

AT is another procedure in the surgical management of the overacting IO, especially when accompanied by DVD [1]. In a study, the IOs were transposed to a position 2 to 4 mm anterior to the lateral end of the IR insertion for treatment of overactive IO and the procedure was reported to be effective for elimination of IOOA and for prevention or substantial reduction of DVD [23]. Farvardin and Attarzadeh performed combined resection and AT of the IO on 15 eyes of 9 patients with DVD of 10 PD or more associated with IOOA and they significantly reduced the mean DVD from 16.6 PD to 2.6 PD. Also, IOOA completely disappeared in 12 eyes, and only in 3 eyes, +1 IOOA persisted at one year's follow-up. In their study, the IO was sutured to the sclera at 1 mm anterior to the lateral border of the IR insertion after a 4 mm resection from the distal end [24].

In the present study, we achieved a surgical success in 46 of 49 eyes (93.9%) with AT procedure when an excellent result is deemed as a grade 0 of IOOA and a DVD or hypertropia <5 PD at 6 months postoperatively. After the surgery, in 1 eye (2%), IOOA persisted and required additional surgery, and in 2 eyes (4.0%), antielevation syndrome developed. The patients with antielevation syndrome were orthotropic in the primary position, and no further surgery was performed.

There are some limitations in our study. First, this study has a retrospective design. Second, it does not include controls in each surgical group to make a comparison. Third, the minimum postoperative follow-up time was short, so a longer follow-up is required to determine postoperative results.

In conclusion, this study showed that IOOA may be accompanied by esotropia, exotropia, AHP, pattern strabismus, convergence insufficiency, DVD, facial asymmetry, or nystagmus. The most common coexisting disorder was esotropia. Also, we demonstrated that all of the three surgical methods including recession, myectomy, and AT are effective in the surgical management of IOOA with minimum side effects. Our results showed that classifying the patients with IOOA based on the required surgical techniques simplified the surgical management of these patients.

## Figures and Tables

**Table 1:** Demographic and ophthalmologic examination findings of the patients with IOOA.

	Total (*n*: 173)	Primary IOOA (*n*: 142)	Secondary IOOA (*n*: 31)	*P* value
Age (years)	9.15 ± 7.9	7.75 ± 5.96	15 ± 12.05	0.001^a^
Gender				
Male	88	73	15	
Female	85	69	16	0.84^b^
Primary position				
Orthotropia	44	22	22	
Esotropia	93	88	5	
Exotropia	36	32	4	0.001^b^
Spherical equivalent	+1.25 (−5.5 to +9.75)	+1.25 (−5.5 to +9.75)	+0.25 (−1.5 to +5.25)	0.001^a^
IOOA				
Unilateral	83	53	30	
Bilateral	90	89	1	0.001^b^
AHP	51	31	20	0.001^b^
Pattern strabismus	35	34	1	0.019^b^
Convergence insufficiency	19	18	1	0.21^b^

IOOA: inferior oblique muscle overaction; AHP: abnormal head posture; ^a^Mann–Whitney *U* test; ^b^*x*^2^ test.

**Table 2:** The success rates of surgical groups.

	Recession	Myectomy	AT
Eye (*n*)	173	64	49
Successful (*n*)	166	63	46
Unsuccessful (*n*)	7	1	3
Success rate (%)	96	98.4	93.9

AT: anterior transposition; *n*: number.

## Data Availability

The data used to support the findings of this study are included within the article.

## References

[1] Sanjari M. S., Shahraki K., Nekoozadeh S., Tabatabaee S. M., Shahraki K., Aghdam K. A. (2014). Surgical treatments in inferior oblique muscle overaction. *Journal of Ophthalmic & Vision Research*.

[2] Caldeira J. A. (2004). Some clinical characteristics of V-pattern exotropia and surgical outcome after bilateral recession of the inferior oblique muscle: a retrospective study of 22 consecutive patients and a comparison with V-pattern esotropia. *Binocular Vision & Strabismus Quarterly*.

[3] Chang B. L., Yang S. W. (1988). Inferior oblique overaction. *Korean Journal of Ophthalmology*.

[4] Mostafa A. M., Kassem R. R. (2018). Comparative study of unilateral versus bilateral inferior oblique recession/anteriorization in unilateral inferior oblique overaction. *European Journal of Ophthalmology*.

[5] Alajbegovic-Halimic J., Zvizdic D., Sahbegovic-Holcner A., Kulanic-Kuduzovic A. (2015). Recession vs myotomy-comparative analysis of two surgical procedures of weakening inferior oblique muscle overaction. *Medical Archives*.

[6] Lee D. C., Lee S. Y. (2017). Effect of modified graded recession and anteriorization on unilateral superior oblique palsy: a retrospective study. *BMC Ophthalmology*.

[7] Akar S., Gökyiğit B., Yılmaz Ö. F. (2012). Graded anterior transposition of the inferior oblique muscle for V-pattern strabismus. *Journal of American Association for Pediatric Ophthalmology and Strabismus*.

[8] Sekeroglu H. T., Dikmetas O., Sanac A. S., Sener E. C., Arslan U. (2012). Inferior oblique muscle weakening: is it possible to quantify its effects on horizontal deviations?. *Journal of Ophthalmology*.

[9] Goncu T., Cakmak S., Akal A., Oguz H. (2016). The effect of anterior transposition of the inferior oblique muscle on eyelid configuration and function. *Indian Journal of Ophthalmology*.

[10] Wilson M. E., Parks M. M. (1989). Primary inferior oblique overaction in congenital esotropia, accommodative esotropia, and intermittent exotropia. *Ophthalmology*.

[11] Chang Y. H., Ma K. T., Lee J. B., Han S. H. (2004). Anterior transposition of inferior oblique muscle for treatment of unilateral superior oblique muscle palsy with inferior oblique muscle overaction. *Yonsei Medical Journal*.

[12] Yumuşak E., Yolcu Ü, Küçükevcilioğlu M., Diner O., Mutlu F. M. (2016). Outcomes of unilateral inferior oblique myectomy surgery in inferior oblique overaction due to superior oblique palsy. *Turkish Journal of Ophthalmology*.

[13] Sekeroglu H. T., Turan K. E., Uzun S., Sener E. C., Sanac A. S. (2014). Horizontal muscle transposition or oblique muscle weakening for the correction of V pattern?. *Eye*.

[14] Dickmann A., Parrilla R., Aliberti S. (2012). Prevalence of neurological involvement and malformative/systemic syndromes in A- and V-pattern strabismus. *Ophthalmic Epidemiology*.

[15] Li Y., Ma H., Zhao K. (2016). Effects of bilateral superior oblique “hang-back” recession in treatment of A-pattern strabismus with superior oblique overaction. *Strabismus*.

[16] Shipman T., Burke J. (2003). Unilateral inferior oblique muscle myectomy and recession in the treatment of inferior oblique muscle overaction: a longitudinal study. *Eye*.

[17] Parks M. M. (1971). A study of the weakening surgical procedures for eliminating overaction of the inferior oblique. *Transactions of the American Ophthalmological Society*.

[18] Rajavi Z., Molazadeh A., Ramezani A., Yaseri M. (2011). A randomized clinical trial comparing myectomy and recession in the management of inferior oblique muscle overaction. *Journal of Pediatric Ophthalmology & Strabismus*.

[19] Parks M. M. (1972). The weakening surgical procedures for eliminating overaction of the inferior oblique muscle. *American Journal of Ophthalmology*.

[20] Min B.-M., Park J.-H., Kim S.-Y., Lee S.-B. (1999). Comparison of inferior oblique muscle weakening by anterior transposition or myectomy: a prospective study of 20 cases. *British Journal of Ophthalmology*.

[21] Edwards W. C., Hess J. B. (1982). Inferior oblique surgery. *Annals of Ophthalmology*.

[22] Davis G., McNeer K. W., Spencer R. F. (1986). Myectomy of the inferior oblique muscle. *Archives of Ophthalmology*.

[23] Mims J. L., Wood R. C. (1989). Bilateral anterior transposition of the inferior obliques. *Archives of Ophthalmology*.

[24] Farvardin M., Attarzadeh A. (2002). Combined resection and anterior transposition of the inferior oblique muscle for the treatment of moderate to large dissociated vertical deviation associated with inferior oblique muscle overaction. *Journal of Pediatric Ophthalmology and Strabismus*.

